# Describing vancomycin serum levels in pediatric intensive care unit (ICU) patients: are expected goals being met

**DOI:** 10.1186/s12887-019-1602-8

**Published:** 2019-07-18

**Authors:** Talita Muniz Maloni, Talita Rantin Belucci, Sandra Regina Malagutti, Guilherme Henrique Campos Furtado

**Affiliations:** 1 Pharmacist Hospital Israelita Albert Einstein, Avenida Albert Einstein, 627 Morumbi, São Paulo, Brasil 05651-901; 2 Division of Medical Practice, Hospital Israelita Albert Einstein, São Paulo, Brazil; 3 Statistics Department, Instituto Israelita de Ensino e Pesquisa Albert Einstein, Hospital Israelita Albert Einstein, São Paulo, Brazil; 4 Division of Infectious Diseases, Escola Paulista de Medicina, Universidade Federal de São Paulo, São Paulo, Brazil

**Keywords:** Vancomycin serum level, Pediatrics, Pharmacokinetics, Pharmacodynamics

## Abstract

**Background:**

In the pediatric population, infections by methicillin-resistant *Staphylococcus aureus* (MRSA) are associated with significant morbidity and hospital costs. Vancomycin is a glycopeptide antibiotic, widely used for the treatment of serious infections by Gram-positive microorganisms, especially MRSA. It is recommended to keep the serum level of vancomycin between 10 and 20 mg/L, that correlates with AUC/MIC > 400 in adults. This pharmacodynamic target is extrapolated to pediatric patients despite the lack of similar evidence. However, recent studies suggest that serum levels between 7 and 10 mg/L are predictive of reaching the pharmacodynamic target in this population. In spite of widespread use, ideal information about dosage for the pediatric population remains limited.

**Methods:**

A retrospective study was conducted in patients admitted to the Pediatric Intensive Care Unit during the period between January 01, 2008 to December 31, 2014. We investigated variables such as age, positive fluid balance and use of vasoactive drugs on the ability of these patients to achieve the proposed recommended serum level target and the vancomycin serum levels.

**Results:**

Our study showed that only 26% of children reached the 10–20 mg/L serum level whereas the 7–20 mg/L serum level was reached by 51% of patients.

**Conclusions:**

We observed no evidence of a significant association between the inadequacy of serum level and age. The positive fluid balance also had no influence on the vancomycin serum level but patients using vasoactive drugs had a greater serum level adequacy than patients not using vasoactive drugs.

## Background

Infections in intensive care units (ICU) are associated with high morbidity and mortality rates [1]. In the United States, infections caused by methicillin-resistant *Staphylococcus aureus* (MRSA) cause 8% of all hospital-acquired infections, and MRSA infections are associated with an estimated 18,650 deaths annually [2]. In the pediatric population, MRSA infections are also associated with significant morbidity and hospital expenses [3].

The appropriate treatment for severe infections caused by *S. aureus* represents a significant challenge as the therapeutic failure can result in death [1]. Vancomycin is a glycopeptide antibiotic widely used for the treatment of severe infections caused by Gram-positive microorganisms, especially MRSA. Currently, it is considered the first choice for empirical therapy of these infections [4, 5].

According to the consensus recommendations of the Infectious Diseases Society of America (IDSA), American Society of Health System Pharmacists and Society of Infectious Diseases Pharmacists, keeping the area under the curve (AUC)/minimum inhibitory concentration (MIC) > 400 is a suitable target to achieve a successful outcome in the treatment of MRSA infections [6]. This pharmacodynamic target is considered the best predictor of microbiological and clinical outcomes when treating MRSA infections [7]. However, the calculation of the AUC is clinically impractical. Trough serum concentration measured shortly before the fourth dose, i.e. steady state, with a value of 15–20 mg/L, is correlated with an AUC/MIC > 400 in adult patients with MRSA MIC < 1 mcg/mL [6].

These recommendations were made following a review of in vitro studies and animal and human studies. However, they were intended to be used for adult patients. These guidelines for vancomycin therapy and adjustment did not include recommendations for pediatric patients making use of vancomycin in pediatric patients a unique and specific challenge [8, 9].

Recent studies involving the pharmacokinetics and pharmacodynamics of vancomycin in pediatric patients suggest that the trough serum level of approximately 7–11 mg/L is sufficient to achieve an AUC/MIC > 400 in the pediatric population [10, 11].

The pediatric population shows differences in pharmacokinetic parameters in relation to adults and therefore requires individualized and specific doses [12]. Besides, the physiological changes in the percentage of body water and renal clearance can also alter pharmacokinetic parameters such as volume of distribution (Vd) and elimination half-life, which can lead to lower than recommended serum levels [13]. Despite the extensive use of vancomycin, dosage information to optimize therapy needs to be further explored [14]. Low concentrations of vancomycin can result in less effective therapy and increased propensity for bacterial resistance due to the risk of not reaching AUC/MIC > 400 [5].

The goal of this study is to evaluate the results of a protocol of vancomycin utilization in a pediatric Intensive Care Unit (PICU). We analyzed the serum levels determining the frequency of children with target serum levels between 10 and 20 mg/L. In addition, we investigated the influence of age, positive water balance and use of vasoactive drugs on the ability of these patients to achieve a target serum level between 10 and 20 mg/L.

## Methods

This study was conducted in a tertiary care, private hospital in São Paulo, Brazil with 629 beds and approximately 194,000 patient-days yearly and approved by the Institutional Review Board and Ethics Committee of Hospital Israelita Albert Einstein and informed consent was not required.

A retrospective study was conducted from January 01, 2008 to December 31, 2014 in the PICU.

This study describes if inpatients who received vancomycin and had at least one serum trough vancomycin measure. Trough concentrations were defined as values taken within 1 h of the next due dose. The hospital guidelines stipulate that the first trough concentration be measured before the fourth dose. The recommended of all target serum level of vancomycin according to the hospital guidelines is 10 to 20 mg/L.

The data abstracted from the electronic medical record included demographics and clinical data, vancomycin dose and alterations during the entire treatment, duration of treatment, presence of positive fluid balance (water balance), use of vasoactive drugs (dobutamine, dopamine, epinephrine, norepinephrine) on the day of vancomycin serum level collection, etiologic agents isolated in cultures, trough vancomycin levels and outcome status (death was defined as in-hospital mortality). The study included patients older than 28 days and younger than 19 years with a creatinine clearance (ClCr) greater than 50 mL/min who had used vancomycin for over 48 h. *Patients excluded were* newborns patients (post-natal age equal to or less than 28 days old), patients aged 19 years or older, patients treated with vancomycin less than 48 h and patients with a ClCr equal to or less than 50 mL/min during treatment with vancomycin.

### Statistical analysis

The categorical variables were described by absolute and relative frequencies (percentages) and quantitative variables by summary measures as the mean and standard deviation (SD) or median and quartiles, along with minimum and maximum value. For the analysis of possible factors associated with inadequate serum levels, generalized linear models were adjusted with mixed effects in which the identification of the child, of the passage and collection were included in the model as random effects to consider the dependency among this information [15]. Analyses were performed using the SPSS and R programs with the lme4 package [16, 17].

## Results

In the period of study, there were 1899 hospitalizations in the PICU and vancomycin doses were administered to 184 patients (9.7%). Seventy-four patients were not included in the analysis. Thus, we analyzed 110 patients who used vancomycin in the PICU.

In the period of study, 217 collections were performed in 96 of the 110 patients analyzed (87.3%), showing that in 14 (12.7%) of them, no vancomycin trough level was collected. For those patients with serum vancomycin levels, 40 (41.7%) reached a therapeutic level (between 10 and 20 mcg/mL) in at least one collection and 56 (58.3%) did not reach those levels in any collection. The time to reach the therapeutic level from the first dose administered ranged from 0.5 to 17.9 days, with a mean of 2.9 days (IQR: 1.9, 5.3 days).

Table 1 shows the descriptive analysis of inpatient characteristics at the time of hospitalization.

**Table 1:** Characteristics of inpatients (*N* = 110)

Age (years) – n (%)	
Median (IQR)	4.1 (1.2; 10.8)
Age Classification – n (%)	
< 2 years	43 (39.1%)
2–6.9 years	23 (20.9%)
7–12.9 years	25 (22.7%)
13–18 years	19 (17.3%)
Weight (kg) – n (%)	
Median (IQR)	16 (9; 35)
Gender – n (%)	
Female	59 (53.6%)
Male	51 (46.4%)

The PIM II (pediatric index of mortality II) of patients ranged between 0.1 and 27.5, with a median of 1.8 (first quartile 1.0 and third quartile 5.1).

The major diagnoses that appeared most frequently were respiratory failure in 30.0% of patients followed by major post-op surgery in 20.9% of patients.

Most of the patients presented underlying conditions (71.8%), with neurological diseases being most frequent (25.5%). Some patients had more than one underlying conditions: two patients had heart disease and neurological disease, two with respiratory failure and neurological disease, two with neurological and endocrine/metabolic diseases and one with neurological disease and cancer.

In the sample studied, 48.2% (*n* = 53) of the patients had a positive microbiologic culture. The patients had between one and five agents identified. *Staphylococcus aureus* was the most frequent agent followed by *Staphylococcus epidermidis* (Table 2).

**Table 2:** Positive microbiologic cultures of vancomycin serum level collection (*N* = 110)

Microbiologic culture – n (%)	
Negative	57 (51.8%)
Positive	53 (48.2%)
Positive microbiologic culture^a^ – n (%)	
*Staphylococcus aureus*	12 (10.9%)
*Staphylococcus epidermidis*	9 (8.2%)
*Pseudomonas aeruginosa*	8 (7.3%)
*Escherichia coli*	5 (4.5%)
*Enterobacter cloacae*	4 (3.6%)
*Streptococcus pneumoniae*	4 (3.6%)
*Proteus mirabilis*	2 (1.8%)
*Serratia marcescens*	2 (1.8%)
*Staphylococcus haemolyticus*	2 (1.8%)
Others	12 (10.9%)

^a^More than one positive organism for the same microbiologic culture

The number of samples taken for evaluation of vancomycin trough serum levels varied between 0 and 11, being undertaken in 87.3% of the 110 patients evaluated.

The average duration of vancomycin treatment was 8.7 days (first quartile: 4.3 days and third quartile: 13.0 days). Seven patients died during hospitalization representing 6.4% of the sample of 110 patients. The trough levels related to this mortality rate ranged from < 5 to 17.3 mg/L.

The information about the interval between dose administration and vancomycin serum collection, time between the collection and the next dose and time of collection are described in Table 3.

**Table 3:** Interval between dose and vancomycin serum level collection, time between the collection and the next dose and time of collection for the initial treatment (*n* = 96) and all collections (*n* = 217)

	First collection (n = 96)	All the collections (n = 217)
Interval between dose and the next collection (days) – n (%)		
Median (IQR)	2.0 (1.3; 3.3)	2.1 (1.4; 3.5)
Time between the collection and the next dose (minutes) – n (%)		
Median (IQR)	30 (15; 52.5)	32 (15; 48)
Time of collection – n (%)		
Trough (up to 1 h before the next dose)	79 (82.3%)	182 (83.9%)
More than 1 h before the next dose	17 (17.7%)	35 (16.1%)

The median of the initial dose was 40 mg/kg/day, ranging from 30 to 84 mg/kg/day. In relation to all doses administered during treatment with vancomycin, the total daily dose of vancomycin varied from 24 mg/kg/day to 100 mg/kg/day, with a median of 40 mg/kg/day.

Table 4 shows the variation of vancomycin serum levels. The values of vancomycin serum levels varied between < 5 and 31.4 mg/L, with a median of 7.7 mg/L (first quartile < 5 mg/L and third quartile 10.6 mg/L). The serum levels between 10 and 20 mg/L were reached in 26.3% of the collections and the serum levels between 7 and 20 mg/L were reached in 51.6% of the collections. Serum levels of less than 10 mg/L represented 69.1% of all collections.

**Table 4:** Variation of vancomycin serum level according to daily dose (*n* = 217)

Vancomycin serum levels (mg/L) – n (%)	Vancomycin: Daily dose (mg/kg/day)			
	≤ 40 (*n* = 129)	41 to 50 (*n* = 26)	51 to 60 (*n* = 35)	≥ 61 (*n* = 27)
≤ 5	49 (38, 0%)	7 (26, 9%)	13 (37, 1%)	3 (11, 1%)
6 to 9	43 (33, 3%)	7 (26, 9%)	14 (40, 0%)	14 (51, 9%)
10 to 14	24 (18, 6%)	6 (23, 1%)	6 (17, 1%)	6 (22, 2%)
15 to 20	8 (6, 2%)	5 (19, 2%)	2 (5, 7%)	0 (0, 0%)
≥ 21	5 (3, 9%)	1 (3, 8%)	0 (0, 0%)	4 (14, 8%)

Patients receiving dose of ≤40 mg/kg/day presented a serum level < 10 mg/L in 71.3% of the collections, with this same dosage the serum level between 10 and 14 mg/L was reached in only 18.6% of the collections.

Of all vancomycin serum level collections performed (n = 217), only 211 were possible to identify if the patients used vasoactive drugs at the time of vancomycin serum level collection. 89,6% (*n* = 189) did not use vasoactive drugs and 10,4% (*n* = 22) used vasoactive drugs.

Table 5 shows the results of the mixed logistic models that analyzed factors associated with inadequate serum levels of vancomycin. None variable is related to inadequate vancomycin levels. Otherwise, we found that patients using vasoactive drug had the odds of inadequate serum levels reduced by 73%, compared to patients who did not use vasoactive drug.

**Table 5:** Mixed logistical models of serum level inadequacy

	Odds ratio	IC 95%	*P* value
Age (years)	0.96	(0.89–1.03)	0.242
Age < 2 years	1.00		
Age 2–6.9 years	2.12	(0.81–5.58)	0.127
Age 7–12.9 years	0.51	(0.21–1.23)	0.132
Age 13–18 years	0.95	(0.32–2.86)	0.926
WB positive: Yes	0.94	(0.45–1.95)	0.862
Use of VAD: Yes	0.23	(0.08–0.64)	0.005

*CI 95%* 95% confidence interval for the odds ratios, *WB* water balance, *VAD* Vasoactive drugs

## Discussion

Monitoring of vancomycin serum levels is important both to keep track of the toxicity and effectiveness of the treatment. In adults, AUC/MIC > 400 of vancomycin is associated with better clinical and bacteriological response in patients with MRSA infections, and this target is extended to pediatric patients, despite the lack of similar evidence [18]. Although the AUC/MIC > 400 pharmacodynamic parameter is ideal to determine the clinical efficacy of vancomycin, serum concentration can be used as it is a more practical method [19, 20].

Tkachuk S et al, showed that vancomycin target serum levels varied based on the characteristics of the patient, however, for pediatric patients, in general, levels between 6 and 10 mg/L are sufficient to achieve AUC/MIC ≥400 [18]. Frymoyer*et al*, suggest that trough serum levels of 15–20 mg/L are unnecessary to attain AUC/MIC > 400 in the pediatric population, as minimal concentrations of vancomycin between 7 and 10 mg/L were predictive of reaching the pharmacodynamic target of AUC/MIC > 400 in approximately 90% of the simulations carried out with children receiving the dose of 15 mg/kg every 6 h [10]. Our study showed that only 26% of the children reached a serum level of 10–20 mg/L.

Adult guidelines suggest that AUC/MIC > 400 corresponds to a vancomycin serum concentration of 15 to 20 mg/L for treating MRSA infections, however, children rarely reach this serum level. The study conducted by Kishk et al., found that the correlation of the AUC/MIC > 400 was associated with the average concentration of 11.4 mg/L. [11] In our study, we performed the stratification to assess the distribution of vancomycin serum level with values achieved between 7 and 14 mg/L. We observed that 44.7% of patients receiving vancomycin presented serum levels within this therapeutic range.

Considering the Kishik study, there is a greater number of patients in our study who attained a serum level related to the AUC/MIC > 400 target, with 7–14 mg/L result compared to the 15–20 mg/L serum level. In our study this level was only reached by 6.2% of patients. As this pharmacodynamic parameter best predicts the result in treating invasive infections and the calculation of the AUC is not practical from a clinical standpoint, the common practice is the assessment of vancomycin serum concentrations to monitor the adequacy of the dosage.

The pharmacokinetics of vancomycin differ in pediatric patients and studies in children confirm that few patients reach a trough serum level in the range of 15–20 mg/L when using the current recommended doses [11].

In 2009, Frymoyer et al. found that is unlikely that a vancomycin dose of 40 mg/kg/day in children can reach the recommended pharmacodynamic target of AUC/MIC > 400 for invasive infections by MRSA, even when the MIC is 1mcg/mL [8]. In our study, the average initial dose of 40 mg/kg/day proved to be insufficient to achieve the serum level of 10–20 mg/L, for most patients emphasizing the need for a review of the available literature regarding the dose that offers the best pharmacokinetic and pharmacodynamic efficacy.

We observed no evidence of a significant association between serum level adequacy (10–20 mg/L) and age. A previous study, which evaluated pharmacokinetic and pharmacodynamic parameters of vancomycin in critically ill children also did not demonstrate the influence of age on the parameters evaluated [13]. However, Gordon et al, found significant lower levels of vancomycin in children under the age of six, even using similar doses as those administered to older patients [21]. Madigan et al observed the effect of age and weight on serum concentrations of vancomycin in pediatric patients. The authors suggest for vancomycin’s prescription in pediatric patients should also consider age and weight. The age classification for our study was based in the Madigan et al. study [22].

Another study that evaluated the influence of weight and age in vancomycin serum levels in children also showed that patients aged between two and five years presented initial serum levels below those proposed [2]. The fact that newborns, infants, children and adolescents present distinct physiological development could justify the association between age and lower serum levels. However, due to the pharmacokinetic variability in pediatric patients it is not possible to claim that the effects of maturity on the disposition of drugs are consistent within each age group [12].

In our study, we classified the age groups as < 2 years, 2–6 years, 7–12 years and 13–18 years to assess whether the inadequacy of serum levels was specific of any age group. However, none of the age group showed to be related to inadequate serum levels. In previous studies, the relationship of serum levels according to age led to the dosage recommendations based on the age of the child. Hoang et al., suggest that for patients from one to five months to 13 to 18 years of age, a dose of 60 mg/kg per day would be appropriate for achieving target levels. For patients aged 6 months to 12 years of age, a dose of 70 mg/kg/day would be appropriate [23]. McCabe et al., suggest that in order to achieve a vancomycin serum level above 10 mg/L, the dose for pediatric patients should be according to the age group: 1 month to 2 years: 95 mg/kg/day, 2–12 years: 88 mg/kg/day and 12–18 years: 75 mg/kg/day [24]. Le et al., showed that reaching the AUC/MIC ≥400 target showed a variation according to age, and on that basis, the vancomycin dose of 60 mg/kg/day was ideal for individuals ≥12 years and 70 mg/kg/day for those < 12 years of age [14].

Two important factors that can hinder the serum concentration of antimicrobials are related to the increased volume of distribution and increased renal clearance caused by the intravenous administration of fluids and vasoactive drugs. Often, multiple conditions that can influence the pharmacokinetics are present at the same time, thus excessively complicating the prediction of adequate serum concentrations. In general, the conditions that led to a suboptimal dosage are prevalent. The volume of distribution describes the relationship between the dose and the resulting serum concentration but in conditions where Vd is increased, a reduction in the drug’s serum level concentration is expected [25].

In our study, the positive fluid balance did not show any influence on vancomycin serum levels. In critically ill patients, alterations in physiopathologic conditions that leads to increased capillary permeability, formation of edema, vasodilation and hypotension may result in pharmacokinetics alterations in many antibiotics [26]. Also, the measures taken to reverse this situation such as the administration of large amounts of fluids, make it difficult to interpret the concentrations of vancomycin, the volume of distribution and, subsequently the degree of distribution throughout the tissue [27, 28]. As vancomycin is an antibiotic with a hydrophilic quality, the volume of distribution of vancomycin may be high, while plasma concentrations may be reduce [26]. Katip et al., evaluated pharmacokinetic aspects of vancomycin in patients in the early stage of septic shock and verified that the clearance of vancomycin increased while the volume of distribution did not increase [26]. Once the serum concentration prediction remains difficult in these situations the therapeutic drug monitoring for individual fine tuning of antimicrobial therapy seems most adequate [25].

Only the use of vasoactive drugs (VAD) proved to be a protective factor, since for patients using VAD, the chance of inadequacy of serum levels was 73% lower than patients who did not use VAD. These results are the opposite to what was expected since the use of VAD could modify renal blood flow and glomerular filtration increasing the rates of renal tubular secretion and clearance and, consequently, the elimination of hydrophilic drugs. Future studies are needed to confirm this finding [29].

It is important to note that this study was limited to a single hospital, involving a heterogeneous population of pediatric patients. However, as demonstrated, the administration of vancomycin as well as the monitoring of its use was in agreement with the recommended protocols.

Our results may be useful in the management of the vancomycin dose for pediatric patients guided by serum levels taking into account the frequency of children who reach the target between 10 and 20 mg/L. The study of the relationship between the prescribed dose and serum level attained can contribute to the customization of treatment and monitoring of vancomycin therapy in children, increasing patient safety for a more effective therapy and lower risk of toxicity.

The serum level of 10-20 mg/L is hard to obtain in practice and certain clinical situations may warrant acceptance of lower targets that are more often obtained with current recommended doses.

Our study had some limitations as a single center retrospective study. Namely we could not identify the reason for instituting Vancomycin treatment. Further we did not evaluate efficacy or safety of the treatment including nephrotoxicity and adverse reactions.

## Conclusion

The vancomycin serum level of 10-20mcg/mL was not achieved by most of patients as compared to the therapeutic range of 7-20mcg/mL that was achieved most frequently. According to the severity and location of the infection, the agent involved and the minimum inhibitory concentration of the pathogen, the 7–20 mcg/mL target might be enough for a microbiological and clinical efficacy.

## Data Availability

Confidentially agreements prevent us from sharing the raw data generated during this study. The data available upon request. The corresponding author should be contacted if someone wants to request the data.

## Abbreviations

AUC: Area under the curve
ClCr: Creatine clearance
ICU: Intensive care units
IDSA: Infectious Diseases Society of America
MIC: Minimum inhibitory concentration
MRSA: Methicillin-resistant *Staphylococcus aureus*
PICU: Pediatric intensive care unit
PIM II: Pediatric index of mortality II
VAD: Vasoactive drugs
Vd: Volume of distribution
WB: Water balance
